# A Set of Possible Markers for Monitoring Heart Failure and Cognitive Impairment Associated: A Review of Literature from the Past 5 Years

**DOI:** 10.3390/biom14020185

**Published:** 2024-02-03

**Authors:** Maria Pagano, Francesco Corallo, Piercataldo D’Aleo, Antonio Duca, Placido Bramanti, Alessia Bramanti, Irene Cappadona

**Affiliations:** 1 IRCCS Centro Neurolesi Bonino-Pulejo, Via Palermo, S.S. 113, C.da Casazza, 98124 Messina, Italy; maria.pagano@irccsme.it (M.P.); piercataldo.daleo@irccsme.it (P.D.); antonio.duca@irccsme.it (A.D.); bramanti.dino@gmail.com (P.B.); irene.cappadona@irccsme.it (I.C.); 2 Faculty of Psychology, Università degli Studi eCampus, Via Isimbardi 10, 22060 Novedrate, Italy; 3 Department of Medicine, Surgery and Dentistry, University of Salerno, 84081 Baronissi, Italy; abramanti@unisa.it

**Keywords:** biomarker, heart failure, cognitive impairment

## Abstract

Background: Heart failure is an epidemiologically relevant disease because of the aging population and widespread lifestyles that promote it. In addition to the acute event, it is possible for the disease to become chronic with periodic flare-ups. It is essential to study pathology from a diagnostic and prognostic point of view and to identify parameters for effective monitoring. In addition, heart failure is associated with multiple comorbidities, including cognitive impairment, which is monitored clinically but not through specific biomarkers in these patients. The purpose of this review is to gather the most recent scientific evidence on a few possible biomarkers previously identified for monitoring heart failure and associated cognitive impairment. Methods: We surveyed studies inherent to a set of previously identified markers, evaluating English-language articles from the past five years conducted in adult heart failure patient populations. We used the databases PubMed, Web of Sciences, and Cochrane Library for search studies, and we considered articles published in journals with an impact factor greater than five in the publication year. Results: Among the biomarkers evaluated, a concordant indication for serial measurements for heart failure monitoring emerged only for interleukin-6. For the other markers, there is still little evidence available, which is interesting but sometimes conflicting. Interesting studies have also emerged for biomarkers of cognitive decline assessed in patients with heart failure, confirming the hypotheses of the increasingly studied heart-brain correlation. Conclusion: Certainly, further studies in large populations are needed to identify effective biomarkers for monitoring heart failure and associated cognitive impairment.

## 1. Introduction

Heart failure (HF) is a severe and globally widespread syndrome [1], characterized by the inability of the heart to pump sufficient blood and oxygen to support the metabolic demands of other organs [2]. There can be several causes, including ischemic heart disease, hypertension, valve disease, and myocardiopathy [3]. The aging population is one of the factors contributing to the increased prevalence of heart failure, as older people are more likely to develop cardiovascular diseases [4]. In addition, the burden of comorbidity, i.e., the presence of several medical conditions simultaneously in an individual, can increase the risk of this syndrome [4]. Heart failure is classified according to the percentage of ejection fraction (EF) [5]. EF represents the portion of blood that is pumped by the heart during each contraction. The physiological values of this parameter vary between 50% and 75% [6,7]. If EF is <40%, the heart failure will be at reduced EF (HFrEF); if EF > 50%, we will have decompensation at preserved ejection fraction (HFpEF); if EF is between 41 and 49, we will have decompensation at slightly reduced EF (HFmrEF) [5]. Heart failure can manifest itself mainly in two forms: acute and chronic. The acute form appears suddenly, with signs and symptoms of heart failure [8]. The chronic form presents slowly and chronically; typical symptoms include dyspnea, fatigue, and swollen ankles [9]. Mild cognitive impairment (MCI), which is estimated to be present in more than a quarter of patients with heart failure, often contributes to the significant clinical burden of the disease. The most impaired cognitive domains are attention, memory, working memory, processing speed, and executive functions [10,11,12]. Studies [10,11] showed that 23 to 50 percent of patients had memory problems and 15 to 27 percent had attention disorders. Careful use of biomarkers could help improve the management of heart failure patients and further improve their prognosis [4].

According to the World Health Organization, a biomarker is any substance, structure, or process that can be measured in the body or its products and that can influence or predict the incidence of outcomes or diseases [13]. Ibrahim and Januzzi [14] summarized the characteristics of an ideal biomarker of HF in five points: (I) the method by which a biomarker is judged should be thorough; (II) the tests used to measure the biomarker should be robust; (III) the biomarker should reflect an important pathophysiological pathway involved in the pathological process of heart failure; (IV) the biomarker should provide information other than that already available through routine physical examinations and laboratory evaluations; (V) the biomarker should contribute to clinical judgement to understand the diagnosis, prognosis, or management of heart failure. A growing number of damage, remodeling, and neurohormonal activation proteins have been discovered, the measurements of which could provide important information on heart failure. Some, such as B-type natriuretic peptide (BNP) and N-terminal proBNP (NT-proBNP), are well validated and established in their use in diagnosis, but serial measurements for titration of drug therapy are not indicated [4]. Biomarkers make it possible to predict the development of signs and symptoms, identify patients with subclinical disease, aid in the diagnosis of heart failure, predict disease trajectories, guide therapeutic management, or act as surrogate endpoints [15]. In addition, regular monitoring of biomarkers allows doctors to evaluate the effectiveness of treatment, adapt therapies according to the patient’s response, and detect complications at an early stage. This will provide important prognostic indications, helping to predict the risk of adverse events and disease flare-ups [16]. Regular monitoring of biomarkers is, therefore, a key practice in the management of heart failure. There are several studies in the literature on biomarkers that evaluate and monitor heart failure, but there is little research examining cognitive biomarkers in patients with this syndrome. Therefore, the aim of this review is to evaluate potential biomarkers for use in monitoring heart failure and associated cognitive impairment.

## 2. Materials and Methods

To evaluate new possible markers for use in heart failure monitoring, we analyzed studies conducted in the past 5 years on some previously chosen biomarkers. Because cognitive impairment is not routinely assessed by biomarkers in patients with heart failure, we also examined possible biomarkers used for this purpose in patients with heart failure. We included articles related to studies on heart failure in adult patients published in English in journals with an impact factor (IF) greater than 5 in the year of article publication. We chose studies in adult patients to analyze biomarkers already used in monitoring programs and therefore replicable. We selected the past five years to review the most recent evidence on heart failure monitoring. In the end, we also decided to screen based on the criterion of IF > 5 as an indicator of disclosure and dissemination to experts in the field because this parameter is dependent on citations. The evaluation of the impact factor was performed via Journal Citation Reports. Exclusion criteria were non-English language, studies conducted only in animals, studies that evaluated only the effects of specific drug treatments on biomarkers, or studies that were conducted in people with heart failure with specific comorbidities. The search started on 20 September 2023. Searches were conducted on PubMed, the Web of Science, and the Cochrane Library. The search performed for each biomarker included three terms: (i) individual marker name; (ii) cardiology; and (iii) heart failure. The following markers were used as the first term: “3-hydroxybutyrate OR beta-hydroxybutyrate” (3-OHB or β-OHB), “interleukin-6” (IL-6), “pentraxin-3” (PTX3), “nitric oxide” (NO), and “cognitive biomarker”, respectively.

## 3. Results

**Figure 1:**
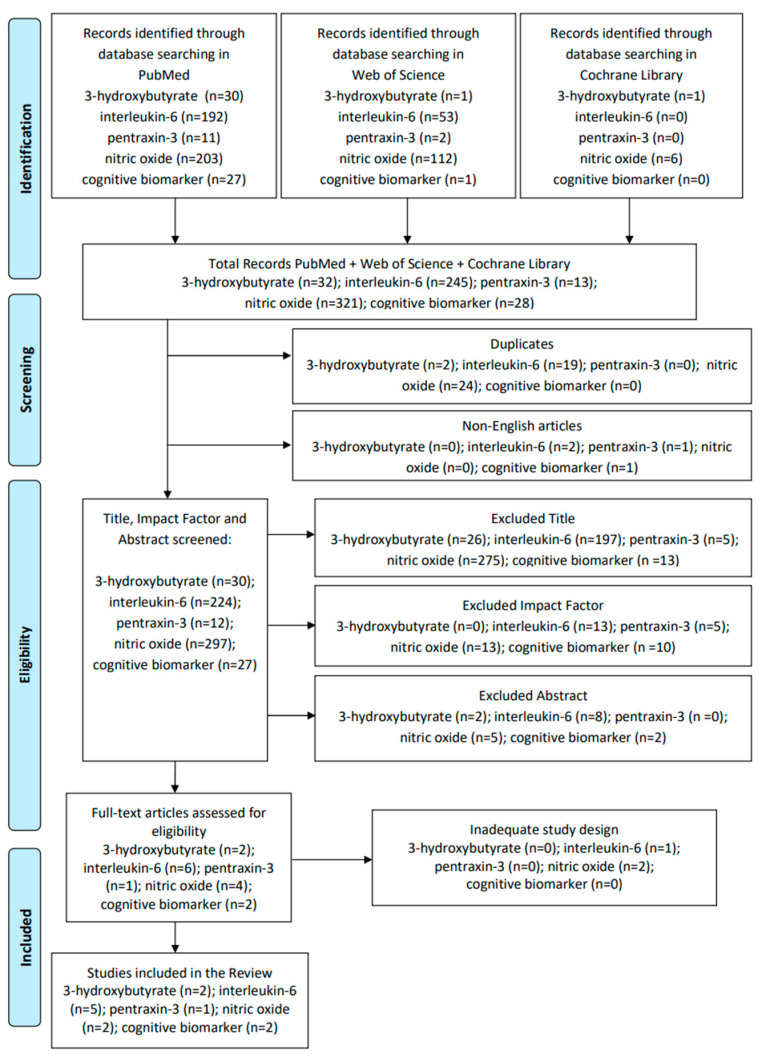
Process of identification, screening, eligibility, and inclusion of the article in the review.

The search carried out produced interesting results, although few studies were eligible for some biomarkers. Details of the process of identification, screening, eligibility, and inclusion in the review are described in Figure 1.

The studies considered were different in sample size, type of study, and analysis tools. In addition, some studies were performed on data from cohorts or subcohorts of other studies. Full details of the studies considered in this review are described in Table 1.

### 3.1. 3-Hydroxybutyrate or Beta-Hydroxybutyrate

Two studies were eligible for this biomarker.

Beta-hydroxybutyrate (β-OHB) is the most abundant circulating ketone body, and its increase has been associated with pathological conditions such as heart failure, other cardiovascular diseases, and chronic kidney disease. Both reviewed studies correlated higher concentrations of ketone bodies with heart failure according to severity.

Voorrips et al. [17] tested ketone bodies by comparing acute phase values with stabilized heart failure values using six measurements (at baseline, after 24 h, after 48 h, after 72 h, after 96 h, and after 30 days of treatment) taken in nonfasting patients. Elevated levels of ketone bodies, particularly acetone, were found in this study. Levels of circulating ketone bodies were higher in patients with acute heart failure.

Flores-Guerrero et al. [18], on the other hand, evaluated the prospective association between beta-hydroxybutyrate and new-onset heart failure in the general population, finding an increased risk in subjects with higher levels of ketone bodies, especially in women.

### 3.2. Pentraxin-3

Only one study was considered for this marker.

Yamamoto et al. [19] analyzed several biomarkers, including pentraxin-3, correlating them with disease outcomes and clinical and instrumental indicators. The correlation of pentraxin-3 with echocardiographic indices revealed associations of this biomarker with abnormal relaxation, increased right ventricular afterload, poor right ventricular systolic function, and increased central venous pressure. In multivariate analyses, PTX-3 was negatively associated with BMI while it was positively associated with atrial fibrillation, sST2 levels, and C-reactive protein. In the prognostic association, PTX-3 did not correlate with cardiovascular death outcomes, all-cause mortality, or re-hospitalizations. Therefore, the results of this study do not elect PTX-3 as a comprehensive biomarker for risk stratification in patients with HF.

### 3.3. Interleukin-6

For the biomarker interleukin-6, five studies were eligible that analyzed the correlation between IL-6 and heart failure.

Chia et al. [21] studied the risk of onset of HF in the general population, and they found that IL-6 levels were significantly correlated with the onset of HFpEF but not HFrEF. Similar results were also found by Alogna et al. [23], although their study was not based on the general population but on patients with HFpEF. They found, in particular, that IL-6 was related to more severe symptoms and a higher BMI. The other studies analyzed associations between IL-6 and natriuretic peptides and cardiovascular outcomes.

Fish-Trotter et al. [20] analyzed circulating levels of IL-6 and NT-proBNP in patients with cardiovascular disease and found that higher levels of IL-6 were associated with higher levels of NT-proBNP at both baseline and follow-up.

Perez et al. [22] also evaluated the correlation between the two parameters with interesting results. Indeed, in Perez’s study, IL-6 levels at 48/72 follow-up were associated with 30- and 180-day mortality, but repeating the analysis by including the NT-proBNP variable remained significantly related to 30-day mortality but not 180-day mortality.

Markousis-Mavrogenis et al. [24] also evaluated the correlation between BNP and IL-6 for the purpose of correlation with death from all causes at 180 days. In particular, this study identified a significant correlation between IL-6 and death from all causes at 180 days, even after adjustment for changes in BNP. In this study, specifically, a doubling of plasma IL-6 concentrations was observed in 17% of patients after 7 days from baseline. Therefore, the authors state that serial measurements of plasma IL-6 concentrations in patients with acute heart failure may have potential prognostic value.

### 3.4. Nitric Oxide (NO)

Schiattarella et al. [29] established the critical role of nitrosative stress in the pathogenesis of HFpEF. Nitric oxide (NO) is a gaseous molecule with a central role in signaling pathways involved in numerous physiological processes, including vasodilation, inflammation, and apoptosis. NO deficiency plays a role in the development of endothelial dysfunction and hypertension [30]. Reduced NO leads to increased passive stiffness in cardiomyocytes [25]. Two studies were selected for this biomarker, although these did not directly analyze nitric oxide but had effects related to it.

Lewsey et al. [26] tested whether endothelium-dependent coronary function (CEF) mediated by NO is impaired in outpatients with stable HFpEF compared with the responses of control subjects. CEF was assessed by coronary MRI based on changes in coronary vasodilation and blood flow in response to isometric hand-holding exercise (IHE). The authors found abnormal CEF in 60% of subjects with HFpEF compared with 0% of age-matched normotensive and hypertensive control subjects.

Momot et al. [25], on the other hand, evaluated some biomarkers, including 3-nitrotyrosine (3-NT), derived from altered NO metabolism, as markers of oxidative/nitrosative stress, finding significantly higher plasma concentrations in patients with HFpEF than in patients with HFrEF. Based on the results, the authors suggest a possible diagnostic and therapeutic value of NO-related markers.

### 3.5. Cognitive Biomarker

For the part about possible monitoring of cognitive impairment by biomarkers, only two studies met the research parameters.

In Dridi et al.’s study [27], through hippocampal biopsy samples from patients with HF and controls, they found that the increased inflammatory response in HFrEF caused intracellular Ca^2+^ leakage mediated by neuronal RyR2, which subsequently affected cognition and memory. This could be attributed to the stress-induced processes of phosphorylation, oxidation, nitrosylation, and depletion of the stabilizing subunit calstabin2.

Lyra et al. [28] found whole brain and regional hypometabolism in heart failure patients with more severe disease undergoing ^18^F-FDG PET/CT. The authors related cerebral glucose hypometabolism to dysfunction of the cerebral blood flow autoregulation (CBF) mechanism, hypoperfusion due to reduced cardiac output, and psychiatric and cognitive comorbidities.

## 4. Discussion

This review was developed with the aim of evaluating additional biomarkers useful in monitoring heart failure and associated cognitive impairment, in addition to biomarkers commonly measured to assess the two medical conditions, respectively. The most common biomarkers for monitoring heart failure are natriuretic peptides, troponin, and inflammatory cytokines [31].

In detail, a recent review states that serial monitoring of N-terminal pro-B-type natriuretic peptide (NT-proBNP), cardiac troponins (cTn), soluble suppression of tumorigenesis-2 (sST2), and galectin-3 levels can help predict clinical outcomes in patients with heart failure. Reductions in NT-proBNP and cTn are predictors of clinical recovery, while increases in cTn, sST2, and galectin-3 predict worse clinical recovery [32].

The most commonly measured biomarkers to assess cognitive impairment are beta-amyloid protein, tau protein, light neurofilaments, and quantitative or topographic biomarkers of neurodegeneration or neuronal damage (CSF t-tau, FDG-PET, or structural MRI) [33,34].

The relationship between the two medical conditions is more and more studied because both diseases are often found together and are related to common risk factors such as population aging and lifestyle. Several authors have shown that vascular contributions to cognitive impairment and dementia (VCID) and conversion to Alzheimer’s disease-related dementia (ADRD) are strongly correlated with vascular disease, inflammation, and decreased cerebral blood flow [35,36,37]. Although the correlation between the two medical conditions is increasingly being analyzed, there are still fragmentary findings on the pathophysiology underlying the coexistence of the two disorders [38]. Marinescu et al. [39] analyzed how age, comorbidities, polytherapies, and other factors affect BNP/NTproBNP levels in geriatric patients (without cognitive impairment) and how this affects the specificity and sensitivity of these biomarkers in identifying heart failure in this category of patients. Bunch et al. [40] analyzed the correlation between vascular dementia and anticoagulant treatments in patients without heart failure. Elahi et al. [41], as part of the SPRINT-MIND study, analyzed the correlation between hypertensive treatments and vascular dementia without further investigating the correlation with HF. Gorelick [34], in his review, analyzes biomarkers of vascular dementia. Hoyer-Kimura et al. [37] analyzed light neurofilaments in mice that were induced by permanent ligation and myocardial infarction. Other authors have found that NfL is elevated in individuals with heart disease [42]. Traub et al. [43] measured serum levels of pTau and NfL in patients with HF, noting that they are affected by age-dependent renal and cardiac dysfunction.

Research is advancing to identify common biomarkers between the two disorders in the human population.

In a most recent study, Wang et al. [44] analyzed the role of brain-derived neurotrophic factor (BDNF) deficiency on the human population, including 50 patients with HF-CI. BDNF is a protein related to cognitive impairment because it regulates synaptic plasticity and excitatory and inhibitory synaptic transmission [45,46,47]. Tissue hypoxia, oxidative stress, and inflammation can produce a decrease in BDNF [48,49], which is also considered a fundamental pathological process of HF. However, Brummel et al. [50] found that markers of systemic inflammation and coagulation measured early during critical illness were not associated with long-term cognitive outcomes.

In addition, since biochemical biomarkers are the result of the expression of genetic biomarkers, much research has been carried out in this regard for the purpose of guidance on the diagnosis of cardiovascular diseases [51,52], drug therapy [53,54], to investigate tissue analysis and biochemical biomarkers [55,56,57], and heart-brain correlation itself [47].

Therefore, the evidence for shedding light on the mechanism linking the two diseases is numerous. The magnitudes of the two disorders analyzed and their multiple manifestations and comorbidities make the research complex and fragmented. This prevented the present review from focusing on biomarkers common to both disorders. However, we believe that the findings emerging in this work will contribute to the evolving analysis of the pathophysiological mechanisms common to the two disorders.

Chronic inflammation plays a crucial role in the pathogenesis of heart failure.

Previous studies have clarified that the heart uses β-OHB as a defense against metabolic stress [58,59], unlike skeletal muscles, which reduce the use of ketone bodies in subjects with HF [60]. Ketone uptake in the hearts of HFpEF and HFrEF patients has been shown to be partially dependent on circulating ketone concentrations [61]. Circulating ketone bodies are higher during heart failure, both during the acute phase and after stabilization, which has led to speculation that neurohormones and natriuretic peptides may influence ketone body concentrations in acute heart failure [62]. The relationship between elevated ketone bodies and HF has been attributed to the change in genetic regulation in individuals with heart failure. This results in a change in the transport and uptake of ketones, which are considered the main fuel of the heart [63]. The Flores-Guerrero [18] study revealed a difference in the association of β-OHB with HF between men and women in the general population, finding that the relationship with HF risk was more significant in women. Indeed, in statistical analysis adjusted for factors such as BMI, cigarette smoking, and alcohol consumption, the relationship in the female sex subjects remained significant, while in the male sex subjects, it lost significance. In contrast, Voorrips [17] longitudinal analysis of all ketone bodies showed that the increase in circulating ketone body concentrations was mainly driven by acetone. Indeed, higher acetone concentrations at baseline were correlated with higher NT-pro BNP values. A similar result was also found by Flores-Guerrero et al. [18]. The authors found that β-OHB was associated with new-onset HFrEF in subjects with higher levels of circulating NT-proBNP at baseline. Future insights into ketone body metabolism in heart failure would be useful in developing new monitoring and treatment protocols.

Pentraxin PTX3 is released into the circulation by vascular or inflammatory cells. Therefore, its presence in the bloodstream is a likely indication of inflammation in the cardiovascular system [64]. Under physiological conditions, PTX3 mRNA is not present in the heart [65]. On the contrary, in the presence of HF and other cardiovascular diseases, PTX3 is expressed in multiple cell types, such as adipose and cardiac cells, in the heart [66,67]. The study considered in our review did not find additional prognostic value for this biomarker. On the contrary, previous studies had correlated higher plasma PTX-3 concentrations with heart failure. Matsubara et al. [68] found a significant association between elevated plasma PTX3 levels and future cardiovascular events in patients with HFpEF. Another study showed that high levels of PTX-3 were associated with a higher risk of cardiovascular events and that levels of the biomarker progressed with severity [69]. Latini et al. [70] also analyzed the role of PTX3 in heart failure, with mixed results. Indeed, the authors found that baseline PTX3 concentration was associated with the risk of cardiovascular mortality or all-cause mortality; however, this association was no longer significant when adjusting for NT-proBNP. In addition, Latini et al. [70] found that basal PTX3 concentration was higher in women than in men, in older patients, and in those with a lower BMI. These results are in agreement with the results of Yamamoto’s study, considered in this review. On the basis of the unclear results for pentraxin 3, further studies evaluating the role of this protein in cardiac decompensation would be useful, as would exploring the possible association between PXT3 and malnutrition, a condition often associated with HF with a negative influence on prognosis.

Fish-Trotter et al. [20] confirmed the hypothesis that inflammation is associated with higher levels of circulating natriuretic peptides (NPs). Indeed, they found elevated IL-6 and NT-proBNP. This occurred not only in heart failure but also in acute respiratory inflammation and sepsis. Since NPs are hormones regulated by feedback loops and have anti-inflammatory properties, this could prove that inflammation can stimulate NP release [71]. BNP and NT-proBNP are the two natriuretic peptides with the most diagnostic value for heart failure [72]. The significant correlation that emerged between IL-6 and HFpEF [22,23] could be an input to continue investigating whether IL-6 constitutes a potential parameter for monitoring HF and a possible therapeutic target. Indeed, the correlation of IL-6 and Nt-proBNP levels with 30-day but not 180-day mortality [22] identifies IL-6 as a possible marker to monitor to identify patients at high risk of rehospitalization. In addition, IL-6 has been associated with greater HF symptom severity and an elevated BMI. The secretion of IL-6 by adipocytes [73] could explain this relationship. In addition, measurements of serial plasma concentrations of IL-6 may indicate patients with persistent inflammation and activation of the cardiosplenic axis, a process that mediates cardiotoxic immune responses [74]. The cardiosplenic axis has an important causal role in heart failure in numerous animal studies, and its activation would be mediated precisely by IL-6 upon solicitation of mononuclear cells. However, Hedayat et al. [75] analyzed IL-6 allele and genotype frequencies in patients with ischemic HF and in a control group and found no association between the −174G/C and +565A/G polymorphisms in the IL-6 gene and ischemic heart failure, while Zheng et al. [76] found a significant correlation between the −572G/C polymorphism of the IL-6 gene and the risk of coronary artery disease, mainly in Asian populations. Therefore, whether the relationship between polymorphisms in IL-6 genes and HF remains controversial, monitoring IL-6 levels can identify patients with elevated levels in each measurement or those in whom increases occur in the interval between two measurements, thus indicating a cardiovascular risk condition.

Nitrosative stress, a disorder of oxygen metabolism, has been shown to be closely associated with cardiovascular disease, including heart failure [77]. In HFpEF, systemic and cardiac inflammation causes activation of the endothelium in the myocardial microcirculation, leading to oxidative stress with uncoupling of endothelial nitric oxide synthase (eNOS) and decreased nitric oxide (NO) production. Another derivative of NO metabolism is 3-nitrotyrosine (3-NT) [78]. The relevance of these mechanisms in the pathogenesis of heart failure was confirmed in the studies of Lewsey and Momot [25,26], analyzed in this review. Other authors have also found similar results previously. Indeed, van Heerebeek et al. [79] also showed that myocardial levels of 3-NT were significantly higher in samples obtained from patients with HFpEF compared with HFrEF. Among the studies included in this review by Lewsey et al. [26], NO-mediated endothelium-dependent coronary function (CEF) was tested by coronary MRI. They found that CEF is impaired in patients with heart failure. However, this tool has limitations: it is performed in health centers, may involve the use of contrast medium, and for these reasons cannot be repeated very frequently. It would be useful to investigate correlations with other biomarkers associated with NO with further studies, including asymmetric dimethylarginine (ADMA), which is an analogue of L-arginine. It has been frequently reported as a new marker of cardiovascular disease risk in numerous studies [80,81,82,83]. Elevated levels of ADMA have been observed in people with hypercholesterolemia, atherosclerosis, hypertension, chronic heart failure, diabetes mellitus, and chronic renal failure. High levels of ADMA inhibit NO synthesis and thus impair endothelial function. ADMA represents an element derived from metabolism present in the bloodstream; hence, it is easily measured.

Clinical and scientific evidence shows that heart failure shares risk factors with dementia processes, and clinical studies link cardiovascular disease and dementia through similar genetic and biochemical profiles and common triggers [84]. In this review, we analyzed the most recent research on possible physiological parameters affecting both cognition and heart failure. Dridi et al. [27] found an association between Ca^2+^ dyshomeostasis, a sign of neurodegenerative diseases, and heart failure. The sympathetic nervous system is continuously activated in patients with HF 25 and is known to be part of an important upstream signaling pathway that alters intracellular Ca^2+^ homeostasis and tightly controls neuronal cell function and survival. Ca^2+^ dyshomeostasis is a common sign of other diseases characterized by progressive muscle and cognitive impairment, such as Huntington’s disease and Parkinson’s disease [85,86]. The association between cerebral glucose hypometabolism and dysfunction of the blood flow autoregulatory (CBF) mechanism was observed in the study by Lyra et al. [28]. Therefore, CBF reduction could be considered a major parameter of heart-brain interconnection in severe heart failure disease. Tran and Wang [87] carried out an in-depth study of the pathways of cardiac glucose metabolism under physiological and pathological conditions. The authors concluded that the heart, at the onset of pathological conditions, undergoes metabolic remodeling by increasing glucose metabolism as a defensive mechanism against injury. However, if the pathological condition persists, repeated metabolic remodeling may be counterproductive, leading to the progression of heart failure. Lyra et al. [28] agree with the concept of a “cardio-cerebral” syndrome [88], according to which bidirectional feedback interactions between the heart and brain would influence the pathophysiology of heart failure. This important interconnection needs further study and investigation.

## 5. Conclusions

Our study did not provide clear evidence for all the biomarkers analyzed or for their use in monitoring HF. This may be attributed to the fact that the biomarkers considered in this review are not specific to the heart. 3-Hydroxybutyrate (3-OHB), pentraxin-3 (PTX3), nitric oxide (NO), and cognitive biomarkers were described in a few studies to determine their validity as parameters for monitoring HF; however, the research provided interesting insights for these indicators. For interleukin-6 (IL-6), multiple studies were selected that agreed in demonstrating a clinic correlation between IL-6 and HF severity, although this was not confirmed by current studies about alleles of the IL-6 gene. Therefore, for IL-6, serial measurements for monitoring HF might be indicated. In the future, continued research in analyzing the pathophysiological mechanisms of HF and combining biochemical, clinical, and instrumental parameters could deepen the fragmented evidence in the literature. To the best of our knowledge, this is the first review to analyze the presence of possible biomarkers for combined monitoring of HF and cognitive impairment. This aspect also needs future studies.

## Figures and Tables

**Table 1:** Study characteristics.

Biomarker	Study	Aim	Sample (N)	Type of Study/Procedure	Results
β-OHB	Voorrips S.N. et al., 2023 [17]	To test if ketogenesis is increased in patients with acute decompensated HF.	79 by the EMPA-RESPONSE-AHF study	Post hoc analysis of patients with acute heart failure: three ketone bodies were measured in nonfasting plasma samples, which were drawn at six timepoints during the treatment phase	Circulating ketone body concentrations were significantly higher during an episode of acute decompensated HF compared with after stabilization.
	Flores-Guerrero J.L. et al., 2021 [18]	To assess whether β-OHB was prospectively associated with HF in the general population.	6134 by the PREVEND study	A prospective population-based cohort study with screening examinations at three different times over a period of about 8 years	High plasma concentrations of β-OHB are associated with an increased risk of HFrEF, particularly in women.
PTX3	Yamamoto M. et al., 2021 [19]	The secondary endpoint was to evaluate the prognostic value of some markers, including PTX-3, in comparison with BNP.	616 by the Ibaraki Cardiac Assessment Study, Heart Failure Registry	A prospective 3-year study of patients with acute decompensated heart failure	This study did not show the additional clinical value of PTX-3 for risk stratification in addition to BNP.
IL-6	Fish-Trotter H. et al., 2020 [20]	Examining correlations between inflammation and NP levels, including associations between circulating levels of IL6 and NT-proBNP.	5481 by the MESA study	A prospective observational study	Increased IL6 was associated with increased NT-proBNP at both baseline and follow-up.
	Chia Y.C. et al., 2021 [21]	To investigate whether a higher plasma level of IL-6 in the general population is associated with an increased risk of developing new-onset HF.	961 by the PREVEND study	Cohort study	IL-6 levels were significantly associated with the development of HFpEF, while the association with HFrEF was not significant.
	Perez A.L. et al., 2021 [22]	Analyze associations between IL-6 and acute decompensation, readmission, and mortality.	883 by the ASCEND-HF study	Cohort substudy	IL-6 at follow-up 48/72 h was independently associated with 30-day mortality but not with 180-day mortality.
	Alogna A. et al., 2023 [23]	To determine the association of IL-6 with changes in cardiac function, congestion, body composition, and exercise tolerance in HFpEF.	374	Cohort substudy	IL-6 levels are commonly found to be elevated in HFpEF, and they are associated with greater symptom severity.
	Markousis-Mavrogenis et al., 2021 [24]	To investigate the prognostic value of serial IL-6 measurements in a cohort of patients with acute HF.	1256 by the PROTECT study	Retrospective cohort analysis	The temporal evolution patterns of IL-6 in patients with acute HF have an additive prognostic value independent of changes in BNP.
Nitric oxide	Momot K. et al., 2023 [25]	To evaluate the circulating levels of markers of inflammation and oxidative/nitrosative stress.	90 (27 HFpEF, 22 HFrEF, and 41 controls)	Observational cohort study	HFpEF is associated with a significantly increased plasma concentration of 3-NT compared to other groups, suggesting that the pathogenesis of HFpEF is associated with oxidative/nitrosative stress.
	Lewsey S.C. et al., 2022 [26]	To test the hypothesis that NO-mediated CEF is impaired in HFpEF patients compared with control subjects by coronary MRI.	32 (10 HFpEF and 22 controls)	Prospective study	~60% of stable HFpEF patients exhibit abnormal CEF.
Cognitive Biomarkers	Dridi H. et al., 2023 [27]	To identify a mechanism for cognitive dysfunction after heart failure in which hyper-adrenergic signaling and transforming growth factor beta activation induce Ca^2+^ leak by RyR2 channels in hippocampal neurons.	13 (9 with HF and 4 control subjects)	Hippocampal biopsy samples were obtained from the Brain Bank	An increased inflammatory response in HFrEF caused intracellular Ca^2+^ leakage mediated by neuronal RyR2 that subsequently affected cognition and memory.
	Lyra V. et al., 2020 [28]	To investigate whole-brain and regional brain glucose metabolism in HF patients and its association with depression comorbidity.	59 (29 subjects with HF and 30 controls)	Cohort study	Heart failure patients with more severe disease showed whole-brain and regional brain hypometabolism in ^18^F-FDG PET/CT.

Legend: HF = heart failure; β-OHB = beta-hydroxybutyrate; HFpEF = heart failure with preserved left ventricular ejection fraction; HFrEF = heart failure with reduced left ventricular ejection fraction; IL-6 = interleukine 6; BNP = brain natriuretic peptide; NT-proBNP = N-terminal pro-brain natriuretic peptide; PTX-3 = pentraxin-3; 3-NT = 3-nitrotyrosine; CEF = endothelium-dependent coronary function; MRI = magnetic resonance imaging; Ca^2+^ = calcium; RyR2 = ryanodine receptor type 2; ^18^F-FDG PET/CT = Fluorine-18 fluorodeoxyglucose positron emission tomography/computed tomography.
